# Serum Retinal and Retinoic Acid Predict the Development of Type 2 Diabetes Mellitus in Korean Subjects with Impaired Fasting Glucose from the KCPS-II Cohort

**DOI:** 10.3390/metabo11080510

**Published:** 2021-08-03

**Authors:** Youngmin Han, Yeunsoo Yang, Minjoo Kim, Sun Ha Jee, Hye Jin Yoo, Jong Ho Lee

**Affiliations:** 1 National Leading Research Laboratory of Clinical Nutrigenetics/Nutrigenomics, Department of Food and Nutrition, College of Human Ecology, Yonsei University, Seoul 03722, Korea; ymhan@yonsei.ac.kr; 2 Institute for Health Promotion, Graduate School of Public Health, Yonsei University, Seoul 03722, Korea; ysyang4647@yuhs.ac (Y.Y.); jsunha@yuhs.ac (S.H.J.); 3 Department of Food and Nutrition, College of Life Science and Nano Technology, Hannam University, Daejeon 34430, Korea; minjookim@hnu.kr; 4 Research Center for Silver Science, Institute of Symbiotic Life-TECH, Yonsei University, Seoul 03722, Korea

**Keywords:** type 2 diabetes mellitus, retinal, retinoic acid, biomarker, disease prediction, liquid chromatography–mass spectrometry

## Abstract

We aimed to investigate whether retinal and retinoic acid (RA), which are newly discovered biomarkers from our previous research, reliably predict type 2 diabetes mellitus (T2DM) development in subjects with impaired fasting glucose (IFG). Among the Korean Cancer Prevention Study (KCPS)-II cohort, subjects were selected and matched by age and sex (IFG-IFG group, *n* = 100 vs. IFG-DM group, *n* = 100) for study 1. For real-world validation of two biomarkers (study 2), other participants in the KCPS-II cohort who had IFG at baseline (*n* = 500) were selected. Targeted LC/MS was used to analyze the baseline serum samples; retinal and RA levels were quantified. In study 1, we revealed that both biomarkers were significantly decreased in the IFG-DM group (retinal, *p* = 0.017; RA, *p* < 0.001). The obese subjects in the IFG-DM group showed markedly lower retinal (*p* = 0.030) and RA (*p* = 0.003) levels than those in the IFG-IFG group. In study 2, the results for the two metabolites tended to be similar to those of study 1, but no significant difference was observed. Notably, the predictive ability for T2DM was enhanced when the metabolites were added to conventional risk factors for T2DM in both studies (study 1, AUC 0.682 → 0.775; study 2, AUC 0.734 → 0.786). The results suggest that retinal- and RA-related metabolic pathways are altered before the onset of T2DM.

## 1. Introduction

Type 2 diabetes mellitus (T2DM) is one of the most prevalent metabolic diseases worldwide and induces many complications [1]. Thus, many studies have focused on the alteration of metabolites prior to T2DM onset for the early prediction of T2DM risk and prevention. Walford et al. [2] reported that metabolites, such as isoleucine, phenylalanine, tyrosine, triacylglycerides (TGs), phosphatidylcholines (PCs), and lysophosphatidylcholines (lysoPCs), could be used as predictive biomarkers up to 13.4 years before the onset of T2DM. The results of a nested case–control cohort study support the idea that isoleucine, L-tyrosine, diacylglycerides (16:0/18:1), lysoPC (19:1), and PC (17:0/18:2) are robustly predictive metabolites of T2DM [3]. Additionally, 2-hydroxyethanesulfonate and PCs containing odd-chain acids (19:1 and 17:0) were identified as novel T2DM-predictive markers in that study.

We previously found that vitamin A (VA)-related metabolites were associated with the incidence of T2DM in impaired fasting glucose (IFG) females, further suggesting their use as early predictive biomarkers of T2DM [4]. VA and its metabolites present in humans are derived from the bioconversion of carotenoids in food or preformed VA supplements. Retinyl esters stored in the liver or adipose tissues are hydrolyzed into retinol when needed and then bound to retinol-binding protein (RBP) to move to peripheral tissues [5]. The enzymatic reaction converts retinol and beta-carotene to a biologically active form, namely, retinoic acid (RA). RA controls the transcription of over 500 retinoid-responsive genes; thus, it is linked with the regulation of adiposity, hepatic steatosis, glucose homeostasis, and any other retinoid-responsive genes that are related to metabolism [6]. To date, the clinical relevance of retinoid metabolism dysregulation related to T2DM is contradictory [7,8,9].

Here, we aimed to investigate whether retinal and RA reliably predict future T2DM development, thereby providing clear insights into retinoid metabolism in T2DM progression. To achieve our goal, we performed targeted metabolomics research via ultra-performance liquid chromatography (UHPLC)-Q Exactive (QE) Orbitrap plus on two independent sets.

## 2. Results

### 2.1. Clinical and Biochemical Characteristics at Baseline

A summary of the overall baseline characteristics of all participants is presented in Table 1. In study 1, 62 subjects were included in the IFG-IFG group and 55 subjects were included in the IFG-DM group. The levels of glucose (*p* = 0.006), ALT (*p* = 0.020), and GGT (*p* = 0.016) were significantly higher in the IFG-DM group than in the IFG-IFG group. Each group was stratified by BMI (nonobese group vs. obese group) (Table 2). In the nonobese subgroup (*n* = 58), the glucose level showed a significant difference between the IFG-IFG group (*n* = 35) and the IFG-DM group (*n* = 23) (*p* = 0.020). On the other hand, in the obese subgroup (*n* = 59), GGT showed a considerable difference between the two groups (*p* = 0.017) (Table 2). No significant difference was observed in the other indicators.

In study 2384 subjects were included in the IFG-IFG group, and 116 subjects were included in the IFG-DM group (Table 1). In the between-group comparison, age (*p* < 0.001), BMI (*p* < 0.001), waist circumference (*p* < 0.001), glucose (*p* < 0.001), TG (*p* = 0.006), total cholesterol (*p* = 0.037), AST (*p* < 0.001), ALT (*p* < 0.001), and GGT (*p* = 0.008) were significantly higher in the IFG-DM group than in the IFG-IFG group. After BMI stratification, age (*p* < 0.001), glucose (*p* < 0.001), and TG (*p* = 0.035) showed a significant difference between the IFG-IFG group (*n* = 218) and the IFG-DM group (*n* = 45) in the nonobese subgroup (*n* = 263). The obese subgroup (*n* = 237) showed a significant difference in age (*p* = 0.017), BMI (*p* = 0.028), waist circumference (*p* = 0.015), glucose (*p* < 0.001), AST (*p* < 0.001), and ALT (*p* < 0.001) between the IFG-IFG group (*n* = 166) and the IFG-DM group (*n* = 71) (Table 2).

### 2.2. Method Validation of UHPLC-QE Orbitrap Plus MS Analysis

The results of our UHPLC-QE Orbitrap plus MS method validation are shown in Table 3. As shown in Table 3, the calibration curves of both metabolites of interest were well fitted linearly. With regard to limit of detection (LOD) and limit of quantitation (LOQ), the amounts of RA detected using UHPLC-QE Orbitrap plus MS were below the LOQ in the majority of the samples. Therefore, the quantity of RA was calculated (relative quantification) for the statistical analysis of the data; the RA peak area was divided by the IS peak area.

### 2.3. Serum Retinal and Retinoic Acid Analysis Using UHPLC-QE Orbitrap Plus MS

**Figure 1:**
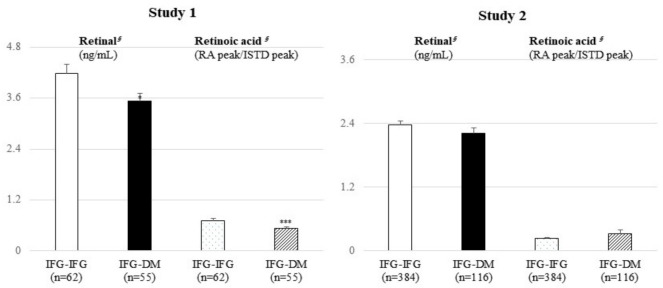
Results of the targeted UHPLC/MS analysis of retinal and retinoic acids between the IFG-IFG and IFG-DM groups. Mean ± standard error (SE). *^§^* tested following a logarithmic transformation. *p*-values were derived from an independent *t*-test; comparison of the baseline levels of retinal and retinoic acids between the IFG-IFG group (IFG at baseline and follow-up) and the IFG-DM group (IFG at baseline but DM developed after the 7-year mean follow-up period). * *p* < 0.05, ** *p* < 0.01, and *** *p* < 0.001 were the results of the within-group comparisons of each group. DM: diabetes mellitus, IFG: impaired fasting glucose, ISTD: internal standard, RA: retinoic acid.

**Figure 2:**
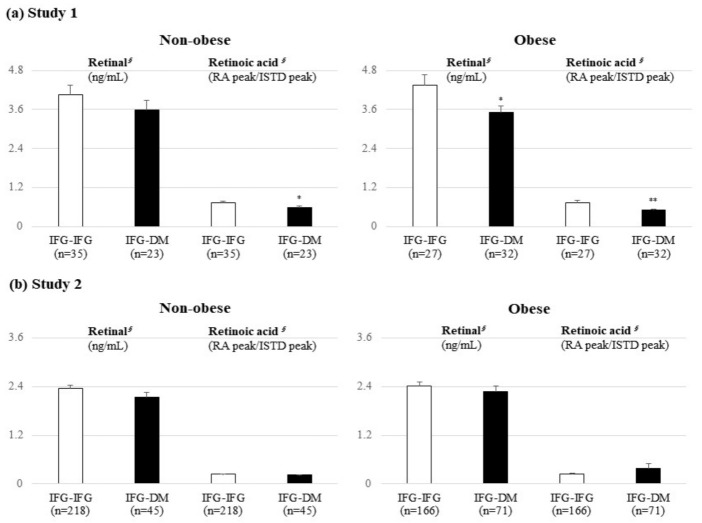
Results of the targeted UHPLC/MS analysis on retinal and retinoic acids between the IFG-IFG and IFG-DM groups stratified by BMI. Mean ± standard error (SE). *^§^* tested following a logarithmic transformation. *p*-values were derived from an independent *t*-test; comparison of the baseline levels of retinal and retinoic acids between the IFG-IFG group (IFG at baseline and follow-up) and the IFG-DM group (IFG at baseline but DM developed after the 7-year mean follow-up period). * *p* < 0.05, ** *p* < 0.01, and *** *p* < 0.001 were the results of the within-group comparisons of each group. DM: diabetes mellitus, IFG: impaired fasting glucose, ISTD: internal standard, RA: retinoic acid.

In study 1, the levels of retinal (*p* = 0.017) and RA (*p* < 0.001) were significantly lower in the IFG-DM group than in the IFG-IFG group (Figure 1). Similar trends were also observed in the obese subgroup; retinal and RA were significantly lower in the IFG-DM group (*p* = 0.030 and *p* = 0.003, respectively) (Figure 2). In the nonobese subgroup, only RA (*p* = 0.023) was markedly lower in the IFG-DM group. On the other hand, no significant difference was confirmed in study 2.

### 2.4. Logistic Regression Analysis

**Figure 3:**
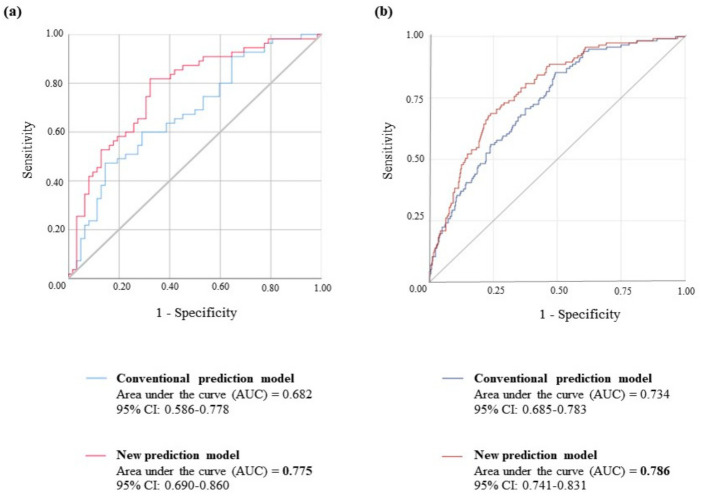
ROC curves for the prediction of T2DM. (**a**) Prediction models with all subjects in study 1 (*n* = 117). The blue line is a prediction model consisting of conventional risk factors (glucose, ALT, GGT) for DM. The pink line is a new prediction model with the metabolites associated with DM development (glucose, ALT, GGT, retinal, retinoic acid). The gray line is a reference line. (**b**) Prediction models with all subjects in study 1 (*n* = 500). The blue line is a prediction model consisting of conventional risk factors (glucose, ALT, GGT) for DM. The red line is prediction model 3 consisting of risk factors (age, BMI, waist circumference, glucose, ALT, GGT, retinal, retinoic acid) for DM. The gray line is a reference line.

Logistic regression analysis was performed to evaluate the independent prediction ability of retinal and RA for T2DM. We constructed each logistic regression model using a combination of conventional risk factors, retinal, and RA (Figure 3). In study 1, an area under the curve (AUC) of 0.682, consisting of traditional risk factors (glucose, ALT, GGT), was significantly improved by adding retinal and RA, with an AUC of 0.775. The predictive ability of the model with retinal and RA (AUC = 0.786) was also stronger than that of a conventional model consisting of glucose, ALT, and GGT (AUC = 0.734) in study 2.

## 3. Discussion

This cohort study confirmed that retinal and RA are valuable biomarkers for predicting T2DM risk in Korean subjects with IFG. In study 1, both retinal and RA were significantly lower in the IFG-DM group than in the IFG-IFG group. From this result, we could infer that subjects with abnormal retinoid metabolism were at high risk of developing T2DM. Although a significant difference in retinal and RA levels between the two groups could not be confirmed in study 2, the predictive power for T2DM was further improved in the prediction model in both studies when adding retinal and RA to the conventional risk factors. Notably, retinal showed significance in the T2DM prediction, even in the real-world validation (study 2).

Our findings are in line with some previous studies. Yan Liu et al. [7,8] showed that the concentration of serum RA determined by enzyme-linked immunosorbent assay (ELISA) was significantly lower in T2DM subjects than in normal glucose tolerance subjects; furthermore, it was inversely associated with ALT, AST, GGT, total cholesterol (TC), and TG, which are traditionally considered T2DM risk factors. Cardiovascular and renal disease, which are well-known complications of DM, also have a deep association with RA. Subjects with coronary artery disease whose circulating RA level was low had a high mortality risk [10]. Agrawal et al. [11] reported that retinoic acid response element (RAR)-α plays a crucial role in renal protection by repairing endogenous RA synthesis. In addition, several epidemiological studies have also revealed that serum carotenoids are negatively correlated with the development of metabolic syndrome. Among Japanese subjects who participated in the longitudinal cohort, people who consumed carotenoids had lower metabolic syndrome incidence and maintained appropriate glucose control ability than people whose carotenoid intake was lower [12]. Similar results were shown for middle-aged and elderly Chinese adults [13]. However, some clinical studies have reported higher serum VA levels in T2DM patients than in nondiabetic controls [9,14]. Additionally, the differences in serum VA levels seen between types of diabetes have not yet been thoroughly explained.

The mechanisms that could explain the lower retinoid levels in the IFG-DM group shown in our study are as follows. The secretion of nonesterified fatty acids (NEFAs) and proinflammatory cytokines increases when body fat increases [15]. Elevated NEFA levels are a hallmark of obesity and T2DM [16,17]. Intracellular NEFAs reduce pyruvate dehydrogenase, phosphofructokinase, and hexokinase II activities while competing with glucose for energy production [18]. Furthermore, increased NEFA levels induce intracellular fatty acid induction, which induces phosphorylation of serine/threonine sites on the insulin receptor, disrupting downstream insulin receptor signaling [19]. Our findings supported these phenomena; although obese subjects in study 1 showed no significant difference in glucose levels between the two groups, the levels of retinal and RA were significantly lower in the IFG-DM group. In other words, retinoid levels may change before a substantial change in glucose occurs. This suggests that retinal and RA can be utilized as essential biomarkers that are comparable to glucose, which is widely used for diabetes diagnosis.

The lower retinoid concentrations shown in the IFG-DM group may indicate abnormal blood glucose control that is closely related to fat accumulation. Some evidence has supported the idea that the action of RA as a ligand of the RAR and retinoid X receptor (RXR) can appropriately control glucose and lipid metabolism [20]. Starkey et al. observed that all-*trans*-RA levels were decreased, accompanied by reduced peroxisome proliferator-activated receptor (PPAR)-β/δ mRNA levels in diabetic mouse kidney tissue [21]. The RAR/RXR dimer binds to the retinoic acid response element (RARE) of target genes, such as glucokinase and SREBP-1c, in the presence of the ligand [22,23]. Glucokinase (Gck) promotes glycogen synthesis and glycolysis [24], and Srebp-1c tends to store lipids rather than utilize them, inducing lipogenesis and cholesterol esterification [25]. It is crucial that retinoids stimulate Srebp-1c and Gck expression by synergizing with insulin. INS-1 cells treated with RA exhibited increased RARE-mediated Srebp-1c expression in a dose-dependent manner [26]. Reduced expression of Gck mRNA in VA-deficient diet-fed rats was recovered with an intraperitoneal injection of RA [27]. Additionally, in rats fed a VA-sufficient diet, an intravenous injection of RA increased hepatic glucokinase expression [28]. Although human studies exploring this mechanism are limited, retinoids and insulin seem to play essential, synergistic roles in glucose and lipid metabolism.

Another possible mechanism is to consider the direct effect of retinoid metabolism on pancreatic β-cells. Although reports still debate the concentration of serum retinoids in T2DM and the link between the two factors, the concentration of retinoids is relatively low in T1DM subjects [14]. From this point of view, the link between insulin secretion and serum retinoids seems to be precise. Pancreatic β-cells, which produce insulin, become dysfunctional and dedifferentiate during diabetes progression [28]. RA signaling during endocrine specification appears to play a critical role in directing pancreatic endocrine cell fate and function [29,30]. Mice fed a VA-deficient diet for eight weeks had reduced β-cell mass, glucose-stimulated insulin secretion, and tissue VA levels [31]. Interestingly, VA-deficient rats treated with VA replication or RA recovered glucose-induced insulin secretion in perifused islets [32]. These results suggest the crucial role of retinoid metabolism in normal glucose metabolism. At this point, we can infer the close relationship between abnormal diabetes metabolism and the decreased retinoid concentrations observed in IFG-DM subjects. We suggest that retinoids directly affect pancreatic β-cells, indirectly regulating insulin secretion and action.

In addition, the control of glucose metabolism associated with retinoids suggested in our study may be the basis for finding targets for T2DM prevention, management, and treatment. RA has been considered a potential therapeutic agent for metabolic diseases based on studies [33]. However, to date, no trials have directly explored the possible treatment effect of RA on T2DM. The representative drugs that can be considered in relation to the function of RA are pharmacological agonists of PPAR and RAR. PPAR-α and RAR-β2 agonists improve dyslipidemia in insulin target tissues, and PPAR-γ agonists, such as thiazolidinediones, improve insulin resistance [34,35]. Therefore, the facts revealed in our study could be key concepts for research on T2DM, including T2DM development.

Our study has several limitations. First, the recovery rate of RA is lower in our metabolomics platform; therefore, further research on RA needs to be performed with the developed analysis methods. Second, there was no information on carotenoid intake in our cohort; it is difficult to draw conclusions about the correlations between carotenoid intake, serum carotenoid, and T2DM occurrence. Despite these limitations, the same direction of the main results shown on two independent sets with large sample sizes is noteworthy. In conclusion, we confirmed the possibility that retinoids, especially retinal, can be used as biomarkers along with glucose for predicting future T2DM risk in Korean subjects with IFG.

## 4. Materials and Methods

### 4.1. Study Subjects

We used two independent sets (studies 1 and 2) to validate the two biomarkers. Study subjects were recruited from the Korean Cancer Prevention Study (KCPS)-II cohort (*n* = 156,701). Detailed characteristics of the KCPS-II cohort were described in our previous study [4,36]. Briefly, the KCPS-II cohort comprises subjects who underwent a routine check-up at 18 health promotion centers in Seoul and Gyeonggi Province, the Republic of Korea, from 2004 to 2013 [37].

**Figure 4:**
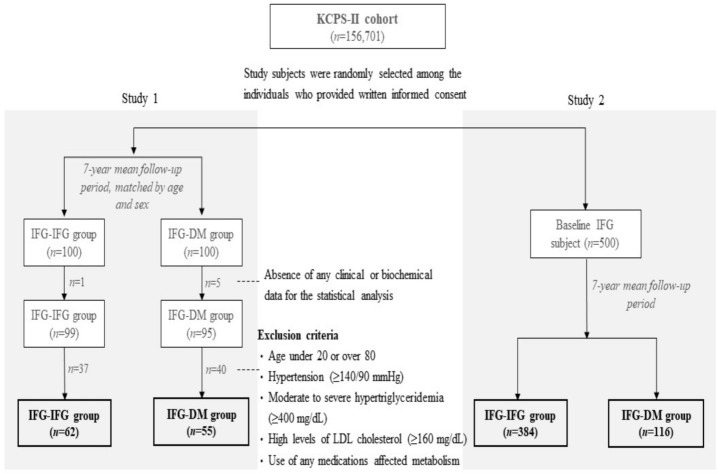
Analysis flow chart. IFG-IFG group: IFG at baseline and follow-up, IFG-DM group: IFG at baseline but DM developed after the 7-year mean follow-up period, DM: diabetes mellitus, IFG: impaired fasting glucose, KCPS: Korean Cancer Prevention Study.

Among all KCPS-II subjects, age- and sex-matched subjects were randomly selected at a ratio of 1:1 for 100 individuals with IFG (100 ≤ serum fasting glucose (mg/dL) < 126) at both baseline and follow-up (IFG-IFG group) and 100 individuals who had IFG at baseline but developed DM (serum fasting glucose ≥ 126 mg/dL) after a 7-year mean follow-up period (IFG-DM group) for study 1. To investigate the predictive capacity of retinal and RA for T2DM, individuals without any clinical or biochemical data for the statistical analysis and who met the exclusion criteria were excluded (Figure 4). As a result, 62 and 55 individuals were included in the IFG-IFG and IFG-DM groups, respectively.

For real-world validation of the two biomarkers (study 2), other participants in the KCPS-II cohort who had IFG at baseline (*n* = 500) were selected without any exclusion criteria. After all the experiments were completed, whether the subjects developed DM was revealed to the researchers. After the exclusion of subjects without any clinical or biochemical data for the statistical analysis, 102 people developed T2DM, and 367 maintained IFG status during the 7-year mean follow-up period (Figure 4).

The Institutional Review Board of Yonsei University Health System reviewed and approved the study, which complied with the Declaration of Helsinki principles (4-2014-1008). We obtained written informed consent for a long-term prospective follow-up from all participants.

### 4.2. Sample Collection and Clinical and Biochemical Assessments

We collected blood samples only at baseline to verify any differences regarding clinical and biochemical assessments between the IFG-IFG and IFG-DM groups before T2DM development. Peripheral venous blood specimens were taken from each subject after a minimum 12 h fasting period. Serum was separated from the specimens via centrifugation, and then, the serum aliquots were stored at −70 °C until further analysis.

To calculate the body mass index (BMI, kg/m^2^), the subjects’ height (cm) and body weight (kg) were measured while they were wearing light clothing. Waist circumference (cm) was measured midway between the lower rib and the iliac crest. Systolic and diastolic blood pressures (mmHg) were measured while the subjects were sitting on a chair after a specified rest period; most health check-up centers used standard mercury sphygmomanometers, but automatic sphygmomanometers were also used at some of the health check-up centers. Levels of fasting glucose, TG, TC, high-density lipoprotein (HDL) cholesterol, low-density lipoprotein (LDL) cholesterol, white blood cell count, aspartate transferase (AST), alanine aminotransferase (ALT), and γ-glutamyl transferase (GGT) were measured using COBAS INTEGRA 800 (Hitachi, Tokyo, Japan) and 7600 (Hitachi, Tokyo, Japan) analyzers. Each laboratory measurement was performed following internal and external quality control procedures specified by the Korean Association of Laboratory Quality Control. The agreement for each biomarker across individual hospitals was high (correlation coefficients ranging from 0.96 to 0.99) [36].

### 4.3. Targeted Metabolic Profiling with UHPLC-QE Orbitrap Plus MS Using Serum Samples

#### 4.3.1. Preparation of Blood Samples

All preparation steps were performed in a dark room to protect the samples from light. One hundred microliters of each serum sample was placed in a 1.5 mL microcentrifuge tube. To precipitate proteins from serum, 275 µL of UPLC-grade acetonitrile (Wako Pure Chemical Industries, Ltd., Osaka, Japan) was added to each sample and then centrifuged (15,000 rpm, 15 min, 4 °C). The supernatant was transferred into a new 1.5 mL microcentrifuge tube and evaporated with nitrogen (N_2_) gas. The dried residue was dissolved in 50% UPLC-grade methanol (J.T. Baker^®^ Chemicals; Avantor Performance Materials, Inc., Randor, PA, USA) for UHPLC-QE Orbitrap analysis. Retinal-d_6_ (Cambridge Isotope Laboratories, Andover, MA, USA) diluted in UPLC-grade ethanol (J.T. Baker^®^ Chemicals; Avantor Performance Materials, Inc., Randor, PA, USA) was used as the internal standard (ISTD). Quality control (QC) samples to be used as a reference to monitor the data quality of a multibatch set were prepared by pooling all samples.

#### 4.3.2. Preparation of Stock Solutions and Standard Samples

To generate calibration curves, stock solutions of the metabolites of interest (retinal and RA) were produced at various concentrations (2.5, 5, 10, 20, 40, and 80 ng/mL), and standard samples were prepared using stock solutions. To make stock solutions, powdered retinal (Sigma-Aldrich, St. Louis, MO, USA) and RA (Sigma-Aldrich, St. Louis, MO, USA) were diluted with UPLC-grade ethanol (J.T. Baker^®^ Chemicals; Avantor Performance Materials, Inc., Randor, PA, USA) and n-hexane (J.T. Baker^®^ Chemicals; Avantor Performance Materials, Inc., Randor, PA, USA), respectively, according to the desired concentrations. Using 100 μL of each stock solution, the standard samples were prepared by following the same protocol used for serum sample preparation described above.

#### 4.3.3. UHPLC-QE Orbitrap Plus MS Analysis

Ten microliters of each prepared serum sample and the standard sample were injected into an Acquity UPLC-BEH-C18 column (2.1 × 50 mm, 1.7 μm; Waters, Milford, MA, USA) equipped with a Thermo UHPLC system (Ultimate 3000 BioRS; Dionex-Thermo Fisher Scientific, Bremen, Germany). The column temperature was maintained at 50 °C. Mobile phases A (0.1% formic acid in LC/MS grade water; Thermo Fisher Scientific, Fair Lawn, NJ, USA) and B (0.1% formic acid in LC/MS grade methanol; Thermo Fisher Scientific, Fair Lawn, NJ, USA) were used with a volumetric concentration change in the range of 0–100% for 22.0 min at 0.4 mL/min.

MS analysis was performed with an electrospray ionization (ESI) source using the QE Orbitrap Plus (Thermo Fisher Scientific, Waltham, MA, USA). The MS conditions were set as follows: spray voltage, 3.5 kV; flow rate of the N_2_ sheath gas, 40 arbitrary units; flow rate of the auxiliary gas, 10 arbitrary units; capillary temperature, 320 °C; S-lens radio frequency level, 50; tube lens voltage, 80 V; and auxiliary gas heater temperature, 300 °C. The MS data were acquired in a parallel reaction monitoring (PRM) assay, which is a technique used for targeted quantification. When the standard samples were analyzed with the PRM method, the conditions of precursor-to-product ion transition *m/z* were as follows: retinal-d_6_: 291.2 → 167.1, retinal: 285.2 → 161.0, and RA: 301.2 → 283.1. Using the conditions as a filter for selecting MS data obtained from the blood samples, metabolite specificity was enhanced and background noise was reduced.

#### 4.3.4. UHPLC-QE Orbitrap Plus MS Method Validation

A calibration curve with five concentration points (2.5, 5, 10, 20, 40, and 80 ng/mL) was generated for each metabolite of interest; the metabolites of interest were measured three times at every concentration. The LOD and LOQ of the metabolites of interest were determined using the following equations: LOD = 3SD/SC and LOQ = 10SD/SC, where SD is the standard deviation of the measurements of the metabolites of interest at each concentration, and SC is the slope of the calibration curve.

Precision was assessed by comparing data measured repeatedly from QC samples. The intra-assay variation was estimated by analyzing six replicates of QC samples, and the inter-assay variation was calculated by analyzing the QC samples on three different days. The precision of the intra- and inter-assay is expressed as the relative standard deviation (%RSD).

For accuracy assessments, 100 µL of spare serum samples was spiked with 25 µL of each stock solution of the metabolites of interest, and then, recovery of the metabolites of interest was calculated using the following equation: recovery (%) = [(A − B)/C] × 100, where A is the amount of the metabolite of interest in 125 µL of the spiked sample, B is the amount of the metabolite of interest in 100 µL of the serum sample, and C is the amount of the metabolite of interest in 25 µL of the stock solution.

## 5. Statistical Analysis

Statistical analysis was conducted using SPSS version 25.0 (IBM/SPSS, Chicago, IL, USA) and SAS 9.4 (SAS Institute Inc., Cary, NC, USA). We performed an independent *t*-test to compare continuous variables between the two groups. Logarithmic transformations were used for skewed variables, and variables with a nonnormal distribution, even after logarithmic transformation, were tested using the Mann–Whitney *U* test. Sex distribution was tested using a chi-squared test. All data are presented as the mean ± standard error (SE), and a two-tailed *p*-value < 0.05 was considered significant. Additionally, we compared the predictive ability for T2DM between the conventional model and the new model with the addition of the two biomarkers using the AUC calculated from logistic regression analysis for each study.

## Figures and Tables

**Table 1:** Baseline clinical and biochemical characteristics of the IFG-IFG and IFG-DM groups in the total population.

	Study 1					Study 2				
	Total (*n* = 117)				*p*	Total (*n* = 500)				*p*
	IFG-IFG (*n* = 62)		IFG-DM (*n* = 55)			IFG-IFG (*n* = 384)		IFG-DM (*n* = 116)		
Age (year)	48.2	±1.41	49.3	±1.23	0.564	48.1	±0.53	52.9	±0.91	<0.001
Male/female, *n* (%)	29 (46.8)/33 (53.2)		26 (47.3)/29 (52.7)		0.957	196 (51.0)/188 (49.0)		54 (46.6)/62 (53.5)		0.458
BMI (kg/m^2^)	25.3	±0.43	26.1	±0.43	0.197	24.8	±0.16	26.1	±0.30	<0.001
Waist circumference (cm)	84.4	±1.17	86.4	±1.01	0.188	83.3	±0.45	87.1	±0.85	<0.001
Systolic blood pressure (mmHg)	124.8	±1.38	124.4	±1.82	0.852	127.6	±0.79	129.1	±1.42	0.347
Diastolic blood pressure (mmHg)	78.2	±1.20	77.8	±0.87	0.757	79.3	±0.63	77.1	±1.13	0.105
Glucose (mg/dL)	109.0	±0.89	113.0	±1.13	0.006 *^∮^*	107.0	±0.39	112.8	±0.62	<0.001
Triglyceride (mg/dL)	140.6	±9.95	149.9	±10.5	0.465 *^∮^*	149.3	±5.39	181.2	±11.1	0.006
Total cholesterol (mg/dL)	191.9	±3.85	191.7	±4.34	0.897 *^∮^*	194.2	±1.76	201.9	±3.37	0.037
HDL-cholesterol (mg/dL)	50.2	±1.08	50.6	±1.32	0.932 *^∮^*	52.4	±0.62	50.2	±1.16	0.094
LDL-cholesterol (mg/dL)	117.7	±3.33	112.1	±4.05	0.202 *^∮^*	118.2	±1.64	122.3	±3.14	0.235
AST (IU/L)	22.9	±1.29	23.9	±1.02	0.204 *^∮^*	23.1	±0.49	28.1	±1.40	<0.001 *^∮^*
ALT (IU/L)	25.2	±2.02	28.8	±1.63	0.020 *^∮^*	25.8	±0.97	37.7	±3.57	<0.001 *^†^*
GGT (IU/L)	30.0	±2.34	36.6	±2.54	0.016 *^∮^*	39.1	±1.99	47.4	±4.94	0.008 *^†^*

Mean ± standard error (SE). Comparisons were conducted between the IFG-IFG group (IFG at baseline and follow-up) and the IFG-DM group (IFG at baseline but developed DM after the 7-year mean follow-up period). Continuous variables were tested using an independent *t*-test, and variables marked with *^∮^* were tested using a logarithmic transformation. Continuous variables with a nonnormal distribution, even after the logarithmic transformation, were tested using a Mann–Whitney *U* test, and *p*-values are marked with *^†^*. Sex distribution was tested using a chi-squared test. DM: diabetes mellitus, IFG: impaired fasting glucose, ALT: alanine aminotransferase, AST: aspartate aminotransferase, BMI: body mass index, GGT: γ-glutamyltransferase, HDL: high-density lipoprotein, LDL: low-density lipoprotein.

**Table 2:** Baseline clinical and biochemical characteristics of the IFG-IFG and IFG-DM groups after stratification by BMI.

Study 1	Nonobese (*n* = 58)					Obese (*n* = 59)				
	IFG-IFG (*n* = 35)		IFG-DM (*n* = 23)		*p*	IFG-IFG (*n* = 27)		IFG-DM (*n* = 32)		*p*
Age (year)	47.1	±2.10	50.5	±1.95	0.268	49.6	±1.75	48.4	±1.59	0.921 *^†^*
Male/female, *n* (%)	20 (57.1)/15 (42.9)		13 (56.5)/10 (43.5)		0.963	9 (33.3)/18 (66.7)		13 (40.6)/19 (59.4)		0.564
BMI (kg/m^2^)	23.1	±0.21	23.4	±0.26	0.328 *^†^*	28.1	±0.61	28.0	±0.47	0.563 *^†^*
Waist circumference (cm)	80.7	±1.08	80.7	±1.22	0.974	89.2	±1.92	90.3	±0.92	0.607
Systolic blood pressure (mmHg)	125.3	±2.06	125.5	±2.77	0.951	124.2	±1.75	123.6	±2.44	0.850
Diastolic blood pressure (mmHg)	79.3	±1.83	77.1	±1.52	0.404	76.9	±1.41	48.3	±1.04	0.429 *^†^*
Glucose (mg/dL)	107.3	±1.08	112.1	±1.80	0.020 *^∮^*	111.0	±1.41	113.6	±1.47	0.219
Triglyceride (mg/dL)	129.7	±10.8	137.8	±16.5	0.859 *^∮^*	154.9	±18.0	158.6	±13.7	0.586 *^∮^*
Total cholesterol (mg/dL)	189.9	±4.59	187.3	±8.13	0.762	194.5	±6.61	194.8	±4.68	0.837 *^†^*
HDL-cholesterol (mg/dL)	51.5	±1.26	51.2	±2.20	0.903	48.6	±1.84	50.3	±1.66	0.501
LDL-cholesterol (mg/dL)	116.2	±3.94	108.6	±6.64	0.302	119.7	±5.76	114.6	±5.12	0.461 *^†^*
AST (IU/L)	21.3	±1.31	21.7	±1.39	0.799 *^†^*	24.9	±2.39	25.5	±1.40	0.156 *^†^*
ALT (IU/L)	23.5	±2.58	24.9	±2.14	0.106 *^†^*	27.4	±3.22	31.6	±2.23	0.081 *^∮^*
GGT (U/L)	29.1	±3.29	28.2	±2.30	0.553 *^∮^*	31.1	±3.31	42.7	±3.71	0.017 *^∮^*
****Study 2****	****Nonobese (*****n*****= 263)**** ****					****Obese (*****n*****= 237)**** ****				
	****IFG-IFG (*n* = 218)****		****IFG-DM (*****n*****= 45)**** ****		***p***	****IFG-IFG (*****n*****= 166)**** ****		****IFG-DM (*****n*****= 71)**** ****		***p***
Age (year)	47.7	±0.72	54.0	±1.50	<0.001	48.8	±0.80	52.2	±1.15	0.017
Male/female, *n* (%)	100 (45.9)/118 (54.1)		17 (37.8)/28 (62.2)		0.407	96 (57.8)/70 (42.2)		37 (52.1)/34 (47.9)		0.416
BMI (kg/m^2^)	22.7	±0.12	22.9	±0.22	0.689	27.5	±0.17	28.2	±0.24	0.028
Waist circumference (cm)	78.5	±0.50	79.2	±1.05	0.555	89.6	±0.54	92.0	±0.78	0.015
Systolic blood pressure (mmHg)	124.7	±1.06	124.3	±2.27	0.877	131.4	±1.11	132.1	±1.75	0.728
Diastolic blood pressure (mmHg)	78.5	±0.84	77.3	±1.89	0.540	80.2	±0.94	77.1	±1.42	0.065
Glucose (mg/dL)	106.3	±0.53	112.4	±0.99	<0.001	107.9	±0.57	113.0	±0.79	<0.001
Triglyceride (mg/dL)	130.2	±5.08	157.0	±13.2	0.035	174.5	±10.2	196.5	±15.9	0.244
Total cholesterol (mg/dL)	192.5	±2.37	198.6	±4.80	0.281	196.5	±2.62	204.1	±4.59	0.133
HDL-cholesterol (mg/dL)	54.4	±0.88	52.3	±1.93	0.331	49.8	±0.81	48.9	±1.44	0.563
LDL-cholesterol (mg/dL)	118.2	±2.07	120.3	±4.81	0.666	118.3	±2.65	123.6	±4.14	0.280
AST (IU/L)	21.8	±0.62	23.5	±1.34	0.269	24.7	±0.76	31.1	±2.05	<0.001 *^†^*
ALT (IU/L)	22.9	±1.17	24.7	±2.40	0.539	29.5	±1.60	45.9	±5.43	<0.001 *^∮^*
GGT (U/L)	32.6	±2.36	43.9	±11.01	0.126 *^∮^*	47.6	±3.28	49.6	±4.13	0.731

Mean ± standard error (SE). Comparisons were conducted between the IFG-IFG group (IFG at baseline and follow-up) and the IFG-DM group (IFG at baseline but developed DM after the 7-year mean follow-up period) in each stratified subset (nonobese and obese). Continuous variables with a normal distribution were tested using an independent *t*-test; the variables with *p*-values marked with *^∮^* were tested following a logarithmic transformation to fit a normal distribution. Continuous variables with a nonnormal distribution, even after logarithmic transformation, were tested using a Mann–Whitney *U* test, and *p*-values are marked with *^†^*. Sex distribution was tested using a chi-squared test. DM: diabetes mellitus, IFG: impaired fasting glucose, ALT: alanine aminotransferase, AST: aspartate aminotransferase, BMI: body mass index, GGT: γ-glutamyltransferase, HDL: high-density lipoprotein, LDL: low-density lipoprotein.

**Table 3:** Results of method validation.

	Retinal		Retinoic Acid	
*R^2^* of calibration curves (linearity)	0.9956		0.9959	
LOQ (ng/mL)	1.63		24.5	
**Precision assessments**				
Intra-assay variation (%RSD)	0.0268		0.0497	
Inter-assay variation (%RSD)	0.0304		0.0645	
**Accuracy assessments**				
	Recovery (%)	SD (%)	Recovery (%)	SD (%)
Recovery at low concentration	93.5	3.83	48.2	6.50
Recovery at high concentration	90.8	7.63	93.1	3.93

For the accuracy assessments, recovery was calculated at two different (low and high) concentrations. For the low concentration, 1 ng/mL and 20 ng/mL retinal and retinoic acid stock solutions were used, respectively, and for the high concentration, 5 ng/mL and 40 ng/mL retinal and retinoic acid stock solutions were used. One hundred microliters of spare serum samples was spiked with 25 µL of each stock solution at different concentrations and then analyzed three times. LOQ: limit of quantification, RSD: relative standard deviation.

## Data Availability

Some or all datasets generated during and/or analyzed during the current study are not publicly available but are available from the corresponding author upon reasonable request.

## References

[1] Khan M.A.B., Hashim M.J., King J.K., Govender R.D., Mustafa H., Al Kaabi J. (2020). Epidemiology of Type 2 Diabetes–Global Burden of Disease and Forecasted Trends. J. Epidemiol. Glob. Health.

[2] Walford G.A., Porneala B.C., Dauriz M., Vassy J.L., Cheng S., Rhee E.P., Wang T., Meigs J.B., Gerszten R.E., Florez J.C. (2014). Metabolite Traits and Genetic Risk Provide Complementary Information for the Prediction of Future Type 2 Diabetes. Diabetes Care.

[3] Shi L., Brunius C., Lehtonen M., Auriola S., Bergdahl I.A., Rolandsson O., Hanhineva K., Landberg R. (2018). Plasma metabolites associated with type 2 diabetes in a Swedish population: A case–control study nested in a prospective cohort. Diabetologia.

[4] Kim M., Jee S., Yoo H., Kang M., Kim J., Lee J. (2017). Serum vitamin A-related metabolite levels are associated with incidence of type 2 diabetes. Diabetes Metab..

[5] O’Byrne S.M., Blaner W.S. (2013). Retinol and retinyl esters: Biochemistry and physiology. J. Lipid Res..

[6] Libien J., Kupersmith M., Blaner W., McDermott M., Gao S., Liu Y., Corbett J., Wall M. (2017). Role of vitamin A metabolism in IIH: Results from the idiopathic intracranial hypertension treatment trial. J. Neurol. Sci..

[7] Liu Y., Chen H., Wang J., Zhou W., Sun R., Xia M. (2015). Association of serum retinoic acid with hepatic steatosis and liver injury in nonalcoholic fatty liver disease. Am. J. Clin. Nutr..

[8] Liu Y., Chen H., Mu D., Fan J., Song J., Zhong Y., Li D., Xia M. (2016). Circulating Retinoic Acid Levels and the Development of Metabolic Syndrome. J. Clin. Endocrinol. Metab..

[9] Rhee E.-J., Plutzky J. (2012). Retinoid Metabolism and Diabetes Mellitus. Diabetes Metab. J..

[10] Liu Y., Chen H., Mu D., Li D., Zhong Y., Jiang N., Xia M. (2016). Association of Serum Retinoic Acid with Risk of Mortality in Patients with Coronary Artery Disease. Circ. Res..

[11] Agrawal S., He J.C., Tharaux P.-L. (2021). Nuclear receptors in podocyte biology and glomerular disease. Nat. Rev. Nephrol..

[12] Sugiura M., Nakamura M., Ogawa K., Ikoma Y., Yano M. (2015). High serum carotenoids associated with lower risk for the metabolic syndrome and its components among Japanese subjects: Mikkabi cohort study. Br. J. Nutr..

[13] Liu J., Shi W.-Q., Cao Y., He L.-P., Guan K., Ling W.H., Chen Y.-M. (2014). Higher serum carotenoid concentrations associated with a lower prevalence of the metabolic syndrome in middle-aged and elderly Chinese adults. Br. J. Nutr..

[14] Erikstrup C., Mortensen O.H., Nielsen A.R., Fischer C., Plomgaard P., Petersen A.M., Krogh-Madsen R., Lindegaard B., Erhardt J.G., Ullum H. (2009). RBP-to-retinol ratio, but not total RBP, is elevated in patients with type 2 diabetes. Diabetes Obes. Metab..

[15] Coppack S.W. (2001). Pro-inflammatory cytokines and adipose tissue. Proc. Nutr. Soc..

[16] Grapov D., Adams S.H., Pedersen T.L., Garvey W.T., Newman J.W. (2012). Type 2 Diabetes Associated Changes in the Plasma Non-Esterified Fatty Acids, Oxylipins and Endocannabinoids. PLoS ONE.

[17] Steinberg G.R. (2007). Inflammation in Obesity is a Common Link Between Defects in Fatty Acid Metabolism and Insulin Resistance. Cell Cycle.

[18] Bergman R.N., Ader M. (2000). Free Fatty Acids and Pathogenesis of Type 2 Diabetes Mellitus. Trends Endocrinol. Metab..

[19] Zhang L., Keung W., Samokhvalov V., Wang W., Lopaschuk G.D. (2010). Role of fatty acid uptake and fatty acid β-oxidation in mediating insulin resistance in heart and skeletal muscle. Biochim. Biophys. Acta (BBA) Mol. Cell Biol. Lipids.

[20] Zhang R., Wang Y., Li R., Chen G. (2015). Transcriptional Factors Mediating Retinoic Acid Signals in the Control of Energy Metabolism. Int. J. Mol. Sci..

[21] Starkey J.M., Zhao Y., Sadygov R.G., Haidacher S.J., Lejeune W.S., Dey N., Luxon B.A., Kane M.A., Napoli J.L., Denner L. (2010). Altered Retinoic Acid Metabolism in Diabetic Mouse Kidney Identified by 18O Isotopic Labeling and 2D Mass Spectrometry. PLoS ONE.

[22] Li R., Zhang R., Li Y., Zhu B., Chen W., Zhang Y., Chen G. (2014). A RARE of hepatic Gck promoter interacts with RARα, HNF4α and COUP-TFII that affect retinoic acid- and insulin-induced Gck expression. J. Nutr. Biochem..

[23] Roder K., Zhang L., Schweizer M. (2007). SREBP-1c mediates the retinoid-dependent increase in fatty acid synthase promoter activity in HepG2. FEBS Lett..

[24] Agius L. (2008). Glucokinase and molecular aspects of liver glycogen metabolism. Biochem. J..

[25] Shao W., Espenshade P.J. (2012). Expanding Roles for SREBP in Metabolism. Cell Metab..

[26] Li R., Chen W., Li Y., Zhang Y., Chen G. (2011). Retinoids synergized with insulin to induce Srebp-1c expression and activated its promoter via the two liver X receptor binding sites that mediate insulin action. Biochem. Biophys. Res. Commun..

[27] Chen G., Zhang Y., Lu D., Li N.-Q., Ross A.C. (2009). Retinoids synergize with insulin to induce hepatic Gck expression. Biochem. J..

[28] Cinti F., Bouchi R., Kim-Muller J.Y., Ohmura Y., Sandoval P.R., Masini M., Marselli L., Suleiman M., Ratner L.E., Marchetti P. (2016). Evidence of β-Cell Dedifferentiation in Human Type 2 Diabetes. J. Clin. Endocrinol. Metab..

[29] Lorberbaum D.S., Kishore S., Rosselot C., Sarbaugh D., Brooks E.P., Aragon E., Xuan S., Simon O., Ghosh D., Mendelsohn C. (2020). Retinoic acid signaling within pancreatic endocrine progenitors regulates mouse and human β cell specification. Development.

[30] Chien C.-Y., Yuan T.-A., Cho C.H.-H., Chang F.-P., Mao W.-Y., Wu R.-R., Lee H.-S., Shen C.-N. (2016). All-trans retinoic acid ameliorates glycemic control in diabetic mice via modulating pancreatic islet production of vascular endothelial growth factor-A. Biochem. Biophys. Res. Commun..

[31] Zhou Y., Zhou J., Zhang Y., Tang J., Sun B., Xu W., Wang X., Chen Y., Sun Z. (2020). Changes in Intestinal Microbiota Are Associated with Islet Function in a Mouse Model of Dietary Vitamin A Deficiency. J. Diabetes Res..

[32] Chertow B.S., Blaner W.S., Baranetsky N.G., Sivitz W., Cordle M.B., Thompson D., Meda P. (1987). Effects of vitamin A deficiency and repletion on rat insulin secretion in vivo and in vitro from isolated islets. J. Clin. Investig..

[33] Olsen T., Blomhoff R. (2019). Retinol, Retinoic Acid, and Retinol-Binding Protein 4 are Differentially Associated with Cardiovascular Disease, Type 2 Diabetes, and Obesity: An Overview of Human Studies. Adv. Nutr..

[34] Wilding J.P.H. (2012). PPAR agonists for the treatment of cardiovascular disease in patients with diabetes. Diabetes Obes. Metab..

[35] Trasino S.E., Tang X.-H., Jessurun J., Gudas L.J. (2015). Retinoic acid receptor β2 agonists restore glycaemic control in diabetes and reduce steatosis. Diabetes Obes. Metab..

[36] Jee S.H., Kim M., Kim M., Kang M., Seo Y.W., Jung K.J., Lee S.J., Hong S., Lee J.H. (2016). Clinical relevance of glycerophospholipid, sphingomyelin and glutathione metabolism in the pathogenesis of pharyngolaryngeal cancer in smokers: The Korean Cancer Prevention Study-II. Metabolomics.

[37] Jee S.H., Batty G., Jang Y., Oh D.J., Oh B.-H., Lee S.H., Park S.-W., Seung K.-B., Kimm H., Kim S.Y. (2014). The Korean Heart Study: Rationale, objectives, protocol, and preliminary results for a new prospective cohort study of 430,920 men and women. Eur. J. Prev. Cardiol..

